# Project Management Monitoring Based on Expected Duration Entropy

**DOI:** 10.3390/e22080905

**Published:** 2020-08-18

**Authors:** Shiva Cohen Kashi, Shai Rozenes, Irad Ben-Gal

**Affiliations:** 1 Department of Industrial Engineering, Tel-Aviv University, Tel-Aviv 6997801, Israel; Shivacohenkashi@yahoo.com; 2 School of Industrial Engineering and Management, Afeka College of Engineering, Tel Aviv 6910717, Israel; rozenes@afeka.ac.il

**Keywords:** project management, information theory, uncertainty

## Abstract

Projects are rarely executed exactly as planned. Often, the actual duration of a project’s activities differ from the planned duration, resulting in costs stemming from the inaccurate estimation of the activity’s completion date. While monitoring a project at various inspection points is pricy, it can lead to a better estimation of the project completion time, hence saving costs. Nonetheless, identifying the optimal inspection points is a difficult task, as it requires evaluating a large number of the project’s path options, even for small-scale projects. This paper proposes an analytical method for identifying the optimal project inspection points by using information theory measures. We search for monitoring (inspection) points that can maximize the information about the project’s estimated duration or completion time. The proposed methodology is based on a simulation-optimization scheme using a Monte Carlo engine that simulates potential activities’ durations. An exhaustive search is performed of all possible monitoring points to find those with the highest expected information gain on the project duration. The proposed algorithm’s complexity is little affected by the number of activities, and the algorithm can address large projects with hundreds or thousands of activities. Numerical experimentation and an analysis of various parameters are presented.

## 1. Introduction

Project management is often defined as the discipline of initiating, planning, executing, and controlling the work of a team to achieve specific goals and meet specific performance measures. It is one of the most applied paradigms in various and diverse areas, such as construction, manufacturing, transportation, education, and software products. A project is defined as a temporary endeavor designed to produce a unique product, service, or result with defined objectives that usually need to be completed within a specified time and under resource and budget constraints [1]. Typically, during the project planning phase, a project is defined by its activities, deliverables, resources, and timelines. From the project plan, the project manager derives the estimated project duration, which is a key factor in measuring the project’s success and often has a significant impact on the project’s costs and revenues [2,3,4]. For example, in many cases, a delay of the project completion date results in fines as well as additional costs. Unfortunately, few projects are executed exactly as planned. In most cases, the actual duration of the project activities differs from the planned duration, impeding an exact estimation of the project completion date.

Enabling a better prediction of a project’s duration and its completion date is thus a major advantage that facilitates better decision making, more accurate risk management, and often leads to higher profitability. This can be achieved by proper monitoring of project activities to provide more information on a project’s status, particularly on the estimated duration and completion date [1].

Continuous monitoring of a project is often impossible practically and might also disrupt the project itself. Therefore, a germane question concerns when to allocate inspection points under a given, often fixed, monitoring budget. Despite the obvious advantages of such a monitoring procedure, few studies have addressed project management monitoring systematically, alongside providing a practical method to find the optimal time points for such inspections. Possibly, one of the reasons for such a gap is that the identification of the optimal inspection policy is a difficult task, as it requires the evaluation of all of the potential project’s scenarios or paths a priori. Depending upon the project duration and the allowed number of inspection points, designing an optimal monitoring plan may require the evaluation of thousands of paths’ options, even for small-scale projects.

The project management literature provides general guidance for determining the control policy, mostly based on practical, non-analytical approaches. Ordinarily, the project milestones are defined as the recommended monitoring points, where the output of a phase and its time frame are compared to the desired output. Another common practice in project monitoring is to set periodic monitoring points (e.g., weekly or monthly) to monitor a project’s progress. To reduce cost and save precious time while allowing the project manager enough information, careful planning is required when defining the frequency and timing of these monitoring points. As we will show in this work, such a periodic monitoring scheme, as well as project milestone monitoring, might be far from optimal when considering the gathered information with respect to the monitoring budget.

This paper presents a numeric analytical approach for identifying a project’s optimal inspection points by using information theory metrics [2], such as entropy reduction and expected mutual information. It aims to measure the information gathered by the outcome of an inspection point about the estimated project duration, which is considered a random variable. We follow the terminology of project management papers and often term these monitoring points as “control points” (henceforth, the terms monitoring point, inspection point, and control point are used interchangeably), although in many times the monitoring is passive (namely, inspecting the ongoing tasks and estimating their completion rates) with no action taken by the project manager to modify the actual time required to accomplish an activity. However, the new information obtained about the activities’ completion rates usually reduces the uncertainty about the project completion time.

The proposed methodology is based on a simulation and optimization model using a Monte Carlo procedure, which replicates the project paths with various expected activities’ durations based on their distributions. For small-scale projects, an exhaustive search can be performed over all potential inspection points, and the potential information gain can be calculated for each point exactly based on the entropy reduction of the project’s completion time. Relying on simulation runs, the times that statistically provide the highest expected information gain are selected as the best candidates for the monitoring points. We show that the proposed algorithm’s complexity is not significantly affected by the number of activities, and thus the algorithm can be applied to large projects with hundreds or thousands of activities. However, the complexity is sensitive to the number of required monitoring points and the project’s total duration as well as the relations between them.

This paper includes numerical experimentation for simulating various types of projects and activities. As mentioned above, previous studies did not directly measure the expected information about the project completion time. Instead, common heuristics for setting the inspection points were used, both in the literature and in the field, such as the ‘maximal number of activities in progress’ and ‘maximal variance of the activities in progress’ policies, and are used here as benchmark methods. We show that, even in relatively small and noncomplex projects, it is not trivial to identify the optimal inspection points, and conventional heuristics can be highly misleading, as they recommend monitoring times whose information gain is far from optimal, or even when no new information can be obtained by such an inspection.

Another interesting observation is that when comparing the same project with different distribution types for its activities’ durations, e.g., by applying the commonly used beta or uniform distribution, the former leads to more coherent and robust policies for the selection of the monitoring points. This observation points to the benefit derived from the additional information that the “most likely” value (the mode) of the beta distribution provides regarding the project duration and its effect on the obtained information. This phenomenon is further addressed when comparing two identical projects, both with similar beta distribution parameters, where one differs from the other only by their mode values in some of the activities. Such a change leads to allocating different monitoring points, demonstrating such a parameter’s importance.

The rest of this paper is organized as follows. Section 2 provides an overview of previous related studies. In Section 3, we present the proposed method for finding the optimal monitoring points, and in Section 4, we discuss the method’s implementation, using examples to further clarify the proposed approach. In Section 5, numerical experiments are presented, and the simulation results are compared to those of other heuristics. Finally, in Section 6, we conclude the paper with a discussion and suggestions for future research.

## 2. Literature Review

The design of a project monitoring system has been mentioned in the literature as a vital part of project management efforts to achieve projects’ objectives [3,5,6,7]. According to these studies, the need for monitoring the project plan arises due to the nature of projects executed in dynamic environments. Among the factors that affect a project’s plan, we find the revision of activities’ duration estimates, delivery failures, changes in technical specifications, technical difficulties, unexpected weather conditions, and labor unrest, to name just a few factors. To react to environmental changes, and as project schedules are often not deterministic, a monitoring system that generates feedback for corrective actions [6] is necessary. Moreover, it is widely recognized that planning and monitoring a project plays a major role in reducing project failures [8].

An important aspect of project control systems is their ability to determine when and where to allocate monitoring points. Although continuous feedback enables modifying plans as soon as deviation occurs, it is costly and increases the possibility of resistance to what may be considered excessive control measures [9]. The project management literature provides general guidance on control policies, mostly based on non-analytical approaches. For example, King and Cleland [10] suggested that the frequency of periodic monitoring depends upon the project size, i.e., small projects should be controlled on a monthly basis, large projects should be controlled on a weekly basis, and medium-size projects presumably should fit in between. Meredith and Mantel [2] recommended that monitoring points be linked to the occurrence of important events, i.e., project milestones, and not only to the calendar; however, the question of how to determine the extent and frequency of control is not addressed in their work. Raz and Erel [5] considered a case wherein *n* monitoring points must be selected during a project’s lifespan. Their objective was to determine the timing of the monitoring points to maximize the total amount of extracted information. Accordingly, the authors focused on the reporting delay, which refers to the amount of time elapsed from the moment an activity commenced up to the time of the measurement. One of their simplifying assumptions was that data on the status of activities that occurred in the past are less informative than data gathered on recent activities. Note that in our study, we do not apply such an assumption, as the project duration and completion time are affected by the past, current, and future activities in the project. Moreover, in the proposed approach, we aim to define a policy that can be implemented at any time point, including at the beginning of the project.

Raz and Erel [5], as well as Tareghian and Salari [11], presented an analytical framework for determining the timing of project control based on maximizing the total amount of information generated by the monitoring points. In particular, Raz and Erel [5] presented an analytical framework for determining the timing of control points, such that to maximize the number and the intensity of the activities that are taking place at that time point. The authors developed an optimal solution procedure based on dynamic programming and a typical s-curve that, for a given number of control points, determines the timing of each control point. Tareghian and Salari [11] proposed a model that first identifies the optimum number of control points and then determines the timing of the points based on the information gathered at each control point using attraction-repulsion mechanisms borrowed from electromagnetism theory. The benefit yielded by the gathered information is then calculated based on the variance of the beta distribution, which is also used in this study. Note that both aforementioned studies relied on simple heuristics to estimate the gathered information rather than using a more precise information theory measure, as done in this study.

Another study that compared the effectiveness of five control timing policies (i.e., ‘equal intervals’, ‘front loading’, ‘end loading’, ‘random’, and ‘no control’) over a project’s duration showed that, while no significant difference was observed between the policies in terms of the required cost to recover from project plan deviations, the ‘end loading’ policy outperformed the others in controlling time overruns [6]. Another quantitative approach proposed by Falco and Macchiaroli [8] is based on the definition of an effort function, which is linearly dependent upon the total number of executed activities within a given period and is inversely related to the total slack time. They assumed that the control intensity is distributed along a bell-shaped curve around the point of maximum effort. This method accords with the conventional industry practice of locating control points at periods wherein the number of activities in progress is maximal and assumes that the maximal information can be extracted at these points.

Sabeghi et al. [12] proposed a dynamic control approach in which they used an adapted facility location model (FLM) coupled with a computer simulation tool and a project crashing model to dynamically determine the timing of the control points in a project’s lifecycle. Weights were assigned to each project activity based on the degree of its importance and criticality. The adapted FLM was used to dynamically determine the timing of the control points. A simulation model measured the project’s progress and predicted possible disruptions. The activity weights were determined by the number of critical paths that contained the activity. The model assumed that the information gathered at the activities’ completion times is more accurate and accordingly considered the time instances at which one or more project activities are completed according to schedule as the potential control points.

The method proposed herein uses concepts of information theory [13], which provides a qualitative and quantitative model of communication depicted as a statistical process. A key measure of information is entropy, which quantifies the information’s expected value. Using entropy, mutual information I(X;Y), which measures the amount of information that one random variable contains about another, can be calculated.

A key element in the proposed approach is the re-evaluation of a given activity’s duration based on the information gathered at a control point. The estimation of a required activity’s duration is key in enabling the project manager to determine the project length. The project management body of knowledge (PMBOK) [1] assumes that the project manager, possibly with the help of experts, can analyze each activity and estimate its anticipated duration. Most project managers use traditional project management techniques such as the project evaluation review technique (PERT) and critical path method (CPM) to plan, schedule, budget, and control the activities associated with projects [2,3]. An important feature of the PERT, which is used in this research, is its ability to address the uncertainty in an activity’s duration [14]. The PERT model contains three time estimates for each activity, namely: optimistic time (Min)—the optimistic time to execute the activity; most likely time (Mode)—the completion time having the highest probability; and pessimistic time (Max)—the longest time required to complete the activity. The beta shape parameters are derived using these three estimates. The PERT model’s stochastic nature suggests that (nearly) any path may be critical, especially if the activity durations are approximated by the distributions, which can extend to infinite (or at least very large) durations.

In many projects, a stage gate control approach is used. This method provides vibrant decision points of go-no go, providing focus in the development process and enabling senior management to review projects in their main phases [15,16,17].

The commonly used control methodology earned value management (EVM) [18,19], originally developed for cost management, cannot be applied in this study, as it forecasts the project schedule by measuring project progress in monetary terms [20]. Moreover, EVM measurements always converge and result in perfect performance, even if the project is behind schedule. In an attempt to overcome these assumptions, earned schedule (ES) was introduced [21,22].

While ES was derived from EVM, instead of measuring the schedule’s performance, it measures the elapsed time. Unfortunately, however, ES cannot be applied in this study, as it relies heavily on the allocated budget to calculate activity performance and does not provide a tool for predicting any future performance based on current measurements and trends. In contrast, this study proposes estimating the activity duration by relying on the relative progress of each activity at the monitoring time while estimating the activities’ distribution parameters. Thus, the proposed approach assumes that the activity duration can be approximated by some probability distribution, while the project manager can estimate its parameters. Note that, even if the project activities are known, the distribution of the overall project duration and thus its completion time cannot be obtained analytically in many cases.

## 3. The Proposed Model

### 3.1. Overview

The proposed model is straightforward in that it uses entropy reduction measures to allocate the project’s inspection points such that they maximize the amount of information on the estimated project’s completion time. The required number of inspection points is determined by the project manager, usually based on a predefined control budget, and provided as an input to the algorithm, along with the project plan that contains a list of activities based on the PERT model [14]. For each activity, the distribution probability, as well as the duration parameters, are defined. Note that, even if the distributions of all of the project’s activities are known a priori, the distribution of the overall project duration, which is determined by the length of the project’s maximal path, is not trivial to obtain analytically. The estimated project duration, denoted by *x*, for a project with m=1,…,M possible paths, where the duration of each path is denoted by $Z_{m},$ is determined by the duration of the critical path ($z_{max}$), i.e., the longest path in the project, as follows:

(1) $$\;x=z_{\max}=\underset{m}{max}\left\{z_{m}\right\}=\underset{m}{max}\left\{\underset{A_{i}\in Z_{m}}{\sum }a_{i}\right\},\;m=1,\ldots ,M;\;a_{i}\in A_{i}.\;$$

where $a_{i}$ is the duration of activity $A_{i}\;$ that belongs to path $Z_{m}$ with a duration $z_{m}$.

It is assumed that the activity durations can be described by some parametric distribution $A_{i}\sim G\left(\theta \right)$. In particular, and without loss of generality, we will use both the uniform and beta distributions to represent those durations. In addition, it is assumed that all activities share the same distribution type, i.e., they are all distributed uniformly or according to the beta distribution.

A centralized project control policy is assumed, by which the project manager receives updates on the ongoing activities and plugs them into the model to re-estimate the project duration. At each control/inspection point, the project manager obtains the actual durations of the completed activities as well as the updated estimated durations of the ongoing activities based on their percentage of completion (i.e., what percentage of the activity is completed at the time of the inspection). However, no new information is obtained on the duration of activities not yet begun. It is further assumed that the information obtained on one activity does not affect that of other activities, and thus, the information measures of various activities are independent.

Concepts of information theory are used in this study to measure the gained information on the estimated project duration at specific inspection points. A basic measure of information gain is the reduction in entropy, which measures a random variable’s uncertainty or its unpredictability, which in this case represents the possible values of the project duration, each of which can be obtained from a set of realization r=1,…,R of the time of the activities that are part of the critical path. These values are either deterministic, if they were executed already, or stochastic, represented by random variables (random variables are often denoted by capital letters, while their realizations are denoted by lower-case letters) if they have not yet occurred. Accordingly, the project duration’s entropy H(X) is:

(2) $$\;H\left(X\right)=-\overset{T}{\underset{\tau =1}{\sum }}p_{\tau }log\left(p_{\tau }\right),\;$$

where

- X is a random variable representing the project duration, taking possible values $X\in \left\{x_{1},\ldots ,x_{max}\right\}$
- R is the total number of realizations measured (e.g., by a Monte Carlo simulator)
- T is the maximal possible project duration (the ‘longest path’ scenario)
- $x_{r}$ is the estimated project duration for a specific realization r, $0\leq x_{r}\leq T,\;1\leq r\leq R$
- $p_{\tau }$ is the probability of X being within the range ∆τ, where ∆τ=[τ−1,τ]; $p_{\tau }=P\left(X\in ∆\tau \right)=P(\tau -1\leq X<\tau )$. For example, $\;p_{8}\equiv P\left(X\in ∆8\right)\equiv P(7\leq X<8)$.

In cases where the variables are independent and identically distributed (i.i.d.), the probability mass function is the following:

(3) $$\;P\left(X=x\right)=P\left(z_{max}=x\right)={\left(F_{Z_{max}}\left(x\right)\right)}^{n}.$$

A critical path’s estimated duration is equal to the sum of the estimated durations of the critical activities, i.e., those that are part of the critical path. The critical path’s variance is equal to the sum of the critical activities’ variances if they are independent. By applying the central limit theorem (CLT), it can be assumed that in a simplified scenario, wherein a project has a single critical path, the project duration follows a normal distribution. Note, however, that such a scenario is often not realistic, as there might be several alternative critical paths. Another approximation is to use the fact that often a project’s activities have estimated mean and variance values. Note that the log-normal distribution is the maximum entropy probability distribution for a random variate X for which the mean, μ, and variance, $\sigma^{2},\;$ of ln(X) are specified. Therefore, it makes sense to use this distribution in the case in which only the expected mean and variance are known or can be estimated. This is justified by considering the central limit theorem in the log domain, where for the multiplication of positive, independent, and identically distributed factors, X follows a log-normal distribution, and Y=ln(X) is normally distributed. Accordingly, the log-normal distribution’s entropy is given by a simple term:

(4) $$H\left(X\right)=\mu +\frac{1}{2}+\frac{1}{2}ln\left(2\pi e\sigma^{2}\right),$$

while Y’s entropy is the following:

(5) $$H\left(Y\right)=ln\left(2\pi e\sigma^{2}\right)$$

Thus, in such a case, one can measure the reduction in the entropy as a function of the variance, which can be better estimated as more realizations are observed. Nonetheless, note that even if the project activities are independent and their variances are known, different paths that share the same activities are not i.i.d. any longer and, in fact, might be highly correlated. Hence, the above uncertainty measures cannot be applied in many real-world projects.

Marshall and Olkin [23] suggested a closed-form distribution for the sum of a few positively correlated Gaussian terms, this result cannot be extended to a large number of terms. Thus, in the general case, such a direct approach cannot be applied to obtain $p_{\tau }$ analytically, as $z_{\max}$ itself is the outcome of a stochastic process that can be determined for specific cases only. In general, no closed-form expression can be derived analytically for the entropy of such a random variable.

A practical approach is to use a Monte Carlo simulation to numerically estimate the project duration’s probability function,$\;{\tilde{p}}_{\tau }$, and its related entropy. This is obtained by simulating enough realizations, estimating the project duration for each realization, and counting the number of instances $c_{\tau }$ for which the project duration is within the range ∆τ. The duration probability is then simply estimated by its frequency as follows:

(6) $$\;{\tilde{p}}_{\tau }=\frac{c_{\tau }}{R}.$$

The estimated entropy of the project duration at a given inspection time t*,* denoted by ${\tilde{H}}_{t}$*,* can be derived by plugging $\;\tilde{p_{\tau }}$ into Equation (2) as follows:

(7) $$\;{\tilde{H}}_{t}\;\equiv {\tilde{H}}_{t}\left(X\right)=-\overset{T}{\underset{\tau =1}{\sum }}{\tilde{p}}_{\tau }log\left({\tilde{p}}_{\tau }\;\right).$$

Thus, the project duration’s entropy varies at different inspection times depending upon the estimated probability function ${\tilde{p}}_{\tau }$, which changes over time (for simplicity of notation, we avoid a more precise notation ${\tilde{p}}_{\tau }^{t}\;$ that denotes the estimated probability function at time t), where the a priori estimated entropy before the project commences, at time t=0, is denoted as ${\tilde{H}}_{0}$. Using the above measures, the information gain about the estimated project duration X  at inspection point t, denoted by ${\tilde{I}}_{t},$ can be measured as the difference between the a priori estimated entropy and the estimated entropy at time t, as follows:

(8) $$\;\;{\tilde{I}}_{t}=\;{\tilde{H}}_{0}-{\tilde{H}}_{t}.$$

In the next section, we present the proposed algorithm, which is used to find the inspection point(s) that yield the maximal information gain. In other words, these are the inspection points that will generate more information for the project manager regarding the estimated project duration. For the selection of a single inspection point, such a procedure is straightforward: Simply calculate ${\tilde{I}}_{t}$ for each potential point t=1,…,T and select the one having the highest value. For the selection of K  inspection points that will lead (jointly) to the maximal ${\tilde{I}}_{t}$ value, a greedy brute force approach is not feasible due to the combinatorial complexity over *K* and *T*, and therefore a suboptimal heuristic is proposed.

### 3.2. The Proposed Algorithm

As indicated above, the proposed approach consists of two main stages: (i) a Monte Carlo simulation procedure and (ii) an optimum-seeking procedure for selecting the best inspection points.

In the Monte Carlo stage, N  simulations are executed, each over *R* realizations of activities’ durations, generating multiple scenarios that represent the state of the project at various time points. N and R are provided as input parameters to the model and determine the level of significance of the best inspection points. In the second stage, an exhaustive search over all possible monitoring points (or combinations of monitoring points in the case of multiple monitoring points) is performed to determine which point(s) provide the maximal information gain on the anticipated project duration. The procedure is executed over all of the realizations in each simulation, and the output is the selected observation point(s) per simulation, usually based on the majority rule. That is, the selected observation point is the one that, in most of the simulated cases, generated the maximal information gain. Thus, this point statistically obtained the highest information gain compared to the other inspection points. Such replications reduce the likelihood of selecting the wrong monitoring point based on a non-representative simulation. Note that the selection of the monitoring point is based on the potential information gain on the project duration and does not take into account possible actions that can reduce the project duration. In addition, it is assumed that the ‘physics’ of the project remain the same, i.e., the project network and the activities’ distribution parameters are not modified as a result of the inspection.

**Figure 1:**
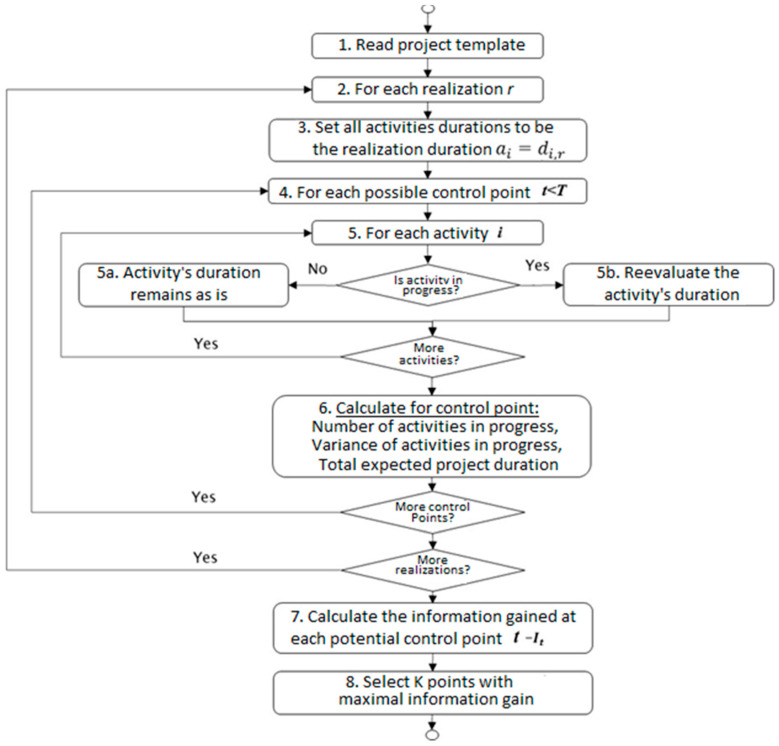
Flow chart of the proposed method.

Figure 1 shows a flow chart of the algorithm for a single simulation run followed by a step-by-step detailed description of the proposed method.

1. The first step is to obtain the project network (using the PERT) and the distribution type with its relevant parameters for each activity.
2. The second step is to obtain the list of realizations, where in each set of realizations, r describes the possible duration of each activity. The model performs Steps 3–6 listed below for each realization r=1,…,R.
3. For each activity i, the activity duration’s initial value,$\;a_{i}$, is defined according to the realization duration, $d_{i,r}$: $a_{i}=d_{i,r}$.
4. An exhaustive search is performed by evaluating every possible monitoring point t  within the range  1≤t≤T, where *T* is the maximal possible length of the project. Steps 5 and 6 below are performed for each potential monitoring point.
5. The project status is re-evaluated by reviewing the status of each activity i in the project plan and identifying the relevant activities in progress. In the event that the activity is still ongoing, the estimated duration is updated (Step 5b in Figure 1) by randomizing a new estimated duration. In contrast (see Figure 1), if the activity has not begun or is already completed, the estimated duration remains as per the initial realization duration or is updated accordingly. Section 3.3 below provides further insights into how the duration is re-evaluated based on the activity’s distribution parameters and the observed status of that activity.
6. Once all activities are reviewed at time t  for realization r, the estimated project duration, $x_{r}$, is calculated based on the new critical path. For benchmark purposes, at this stage, we also calculate the number and sum of the variance terms of the activities in progress. Later, we compare the results of the proposed algorithm to those of heuristics that are based on these measures.
7. Once all possible monitoring points and all realizations are evaluated, the potential information gain, $I_{t},$ is calculated for each potential inspection point t.
8. Based on the above, the K monitoring points with the maximal information gain are selected.

The algorithm’s complexity depends upon the following parameters: N—the number of simulations; T—the maximal project duration; K—the number of required monitoring points; and R—the number of realizations. As mentioned above, the algorithm is exhaustive, and when searching for K  monitoring points, it evaluates all possible combinations to find the one that guarantees the maximum overall information gain. Finding K points with a maximal project duration of T time units requires evaluating $\frac{T!}{K!\left(T-K\right)!}$ such combinations. Hence, the algorithm complexity is $O\left(N\times R\times \frac{T!}{K!\left(N-K\right)!}\right)$. As such, it is clear that T and K are the main drivers that impact the run time.

### 3.3. Re-Evaluating an Activity’s Duration

As described above, when examining a monitoring point t*,* the durations of ongoing activities are re-evaluated. While the exact randomization of the new durations differs from one distribution type to another, it follows the same logic: First, one calculates the percentage (rate) of the completed work, and then this rate is applied to the remaining work. The steps to calculate the new duration of activity i at time t are as follows:

1. Calculate the percentage of completion (poc) of activity i at time  t by dividing the amount of work performed ($w_{i,t}$) defined in time units by the initial estimated duration of activity $a_{i}$ as follows:
  (9) $$\;poc_{i,t}=\frac{w_{i,t}}{a_{i}}.$$
2. Based on the percentage of completed work and the minimal/maximal duration of the activity, $min_{i}$ and $max_{i}$, respectively, re-evaluate the new minimal remaining work, $minRW_{i,t}$, and the new maximal remaining work, $maxRW_{i,t}$, as follows:
  (10) $$\;minRW_{i,t}=\left[1-poc_{i,t}\right]\times min_{i}$$
  (11) $$\;maxRW_{i,t}=\left[1-poc_{i,t}\right]\times max_{i}$$

In the event that a distribution is used to generate the forecasted activity’s duration (as said, we use both the uniform and beta distributions, but other distributions can be applied by following the same logic), recalculate the most likely remaining work, $modeRW_{i,t}$, based on the activity’s distribution mode value, $mode_{i}$, as follows:

(12) $$\;modeRW_{i,t}=\left[1-poc_{i,t}\right]\times mode_{i}.$$

3. Randomly generate a new value for the remaining work, $r_{i}\left(t\right)$, within the following new range:
  (a) In the case where a uniform distribution is used, randomly generate a new value for the remaining work between the minimal and maximal remaining work, and calculate the new duration as follows:
    (13) $$\;a_{i}=w_{i,t}+Rnd\left[minRW_{i,t},maxRW_{i,t}\right].$$
  (b) In the case where a beta distribution is used, first, update the beta parameters based on the amount of work already performed,$\;w_{i}\left(t\right)$, as follows:
    (14) $$\;\;min_{i}=w_{i,t}+minRW_{i,t}$$
    (15) $$\;max_{i}=w_{i,t}+maxRW_{i,t}$$
    (16) $$\;mode_{i}=w_{i,t}+modeRW_{i,t}$$

Based on the new minimal, maximal, and mode values, recalculate the beta shape parameters *α* and *β*. Randomize a new probability value p~U [0,1], and use the beta distribution’s inverse function with the new parameters to generate a new activity duration as follows: $a_{i}=Beta.Inv\left(p,\alpha ,\beta ,min_{i},max_{i}\right)$.

Note that the estimated durations of the ongoing activities are based not only on their completion percentages but also indirectly on the activities completed before that inspection time. The reason is that if a completed activity ended earlier or later than expected, it could affect the completion percentage of future project activities at that inspection time. That is, if a predecessor activity was completed earlier (later) than expected, then more (less) of the ongoing activity that started earlier (later) could be completed at a given inspection time. As a result, the estimation of completing the ongoing activity earlier (later) is updated accordingly. Surely, an earlier/later completed activity that does not belong to the critical path should not necessarily change the completion percentage of later activities.

### 3.4. Searching for Multiple Monitoring Points

The same guiding principles presented above are followed when searching for a number of monitoring points, with a few exceptions that are due to one monitoring point’s effect on the other points. Thus, the information obtained at an inspection point should be measured based on the new information gathered since the last inspection. For example, in the case of two monitoring points $t_{1}$ and $t_{2}$, at $t_{2}$, only the new information obtained since $t_{1}$ should be measured, not all information since t=0, as in the case of a single monitoring point. As explained in step 3 of the proposed algorithm’s flow chart (Figure 1), when searching for a single monitoring point, the duration’s initial value is set to the realization value $d_{i,r}$. Therefore, at the first monitoring point $t_{1}$, this initial realization value,$\;d_{i,r},$ is used to determine the activity’s state; later on, this value is used to randomize a new estimated duration value *v*. When re-evaluating the same activity at the second monitoring point $\;t_{2}$, the updated duration value $a_{i}=v$ is used as a baseline, instead of $d_{i,r}$, while the base realization duration is not used as in the case of a single monitoring point search.

An interesting outcome of the experiments suggests that a possible heuristic may be applied to narrow the search domain and drastically simplify the algorithm run time and its complexity when searching for multiple points. More details on this outcome are shared in Section 5, where we review the results and discuss the conclusions.

## 4. Implementation Example

**Figure 2:**
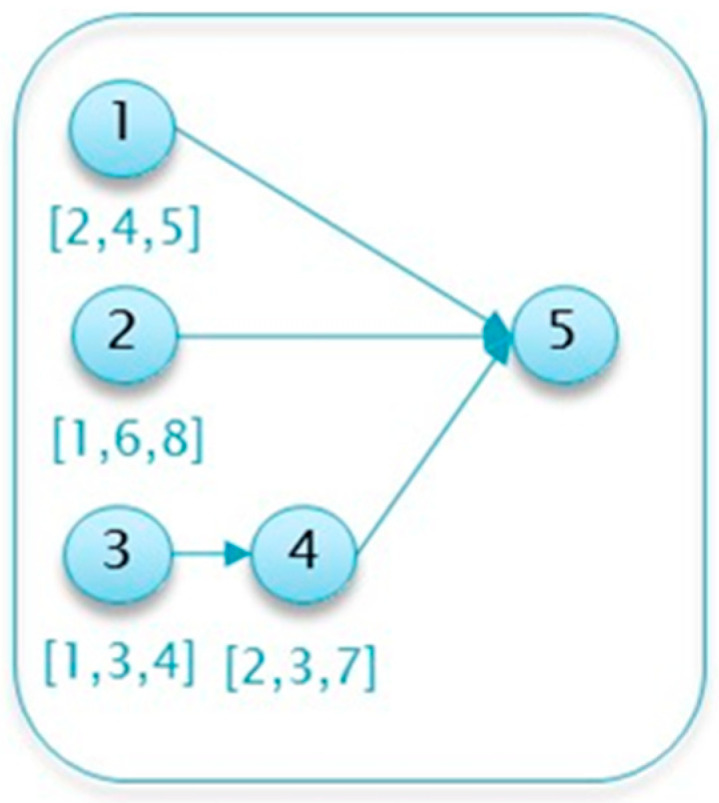
The illustrative running example: the network diagram.

To search for the optimal inspection protocol, conventional project details are required as inputs, i.e., the project’s activities and their characteristics, such as ID, list of predecessor activities, minimal duration, maximal duration, and most likely duration (provided in the case of a beta distribution). In such an implementation, the project and activity details are provided in a tabular format, as shown in the example detailed in Table 1. This project is used as an illustrative running example throughout this section to explain and demonstrate the various algorithm steps. A network diagram depicting this example project is presented in Figure 2.

Similarly, the realizations are also provided in the form of a table of randomized values for each activity’s duration based on the distribution parameters (often in a spreadsheet file). For instance, Activity 1 in the project described in Table 1 is randomized using the underlying beta distribution, i.e., assuming a duration of between 2 and 5 time units and with a most likely (mode) value of 4 time units. Table 2 provides an example of R=10 realizations of the running example project.

Using the realization values, each path’s duration $Z_{m}\;\left(m=1,2,3\right)$ is calculated, as anticipated prior to the project’s commencement (i.e., at time *t =* 0). Table 3 shows the duration of each path ($z_{m}$) in each realization, where the maximal path duration is shaded. The maximal duration over the critical paths determines the project duration, $x_{r}$, which is given in the last column in the table.

From the results presented in Table 3 we can now calculate the project duration’s empirical distribution. For example, in the shortest realizations (with r=3), the project duration is 6.18 time units, thus within the range of [6,7]. Accordingly, the probability of the value lying in this range is estimated empirically as ${\tilde{p}}_{7}=\frac{c_{t}}{R}=0.1$. Similarly, one can estimate the probability of the project’s duration being within the range [7,8] as ${\tilde{p}}_{8}=0.4$; within the range [8,9] as ${\tilde{p}}_{9}=0.4$; and within the range [10,11] as ${\tilde{p}}_{11}=0.1.\;$ Using the estimated probabilities, the base (initial) entropy, ${\tilde{H}}_{0}$, which measures the uncertainty prior to the project initiation, is calculated as follows:

(17) $$\;{\tilde{H}}_{0}=-\underset{i}{\sum }{\tilde{p}}_{t}log\left({\tilde{p}}_{t}\right)=\;-{\tilde{p}}_{7}log\left({\tilde{p}}_{7}\right)-{\tilde{p}}_{8}log\left({\tilde{p}}_{8}\right)-{\tilde{p}}_{9}log\left({\tilde{p}}_{9}\right)-{\tilde{p}}_{11}log\left({\tilde{p}}_{11}\right)=0.518$$

As ${\tilde{H}}_{0}$ is calculated using the initial realization values, it does not change from one simulation run to another as long as there are enough realizations (i.e., the realization values remain the same as long as the underlying distribution is fixed).

Let us now demonstrate how the algorithm evaluates a potential inspection point. Consider, for example, an inspection point at t=4 evaluated for realization r=2. Note that while for some realizations, Activity 1 might still be in progress at t=4, in this realization, the activity duration is $a_{1}=3.77$, and hence, this activity is already completed. Activity 3 is completed as well (as $a_{3}=2.83$), while Activities 2 and 4 are still ongoing. Due to the project dependencies, Activity 5 cannot begin until all of the above activities are completed. Moreover, note that 4 time units of Activity 2 were completed at that time, as well as 1.17 time units of Activity 4. From the amount of work performed on Activity 2 at t=4, $w_{2,4}=4,$ it is possible to derive the percentage of work completed,$\;poc_{2,4}=\frac{w_{2,4}}{a_{2}}=\frac{4}{7}$, which is used to calculate the minimal, maximal, and most likely remaining work, as indicated in Equations (14)–(16), as follows:

$$minRW_{2,4}=\left[1-poc_{2,4}\right]\times min_{2}=\left(1-\frac{4}{7}\right)\times 1=\frac{3}{7}$$

$$maxRW_{2,4}=\left[1-poc_{2,4}\right]\times max_{2}=\left(1-\frac{4}{7}\right)\times 8=3\frac{3}{7}$$

$$modeRW_{2,4}=\left[1-poc_{2,4}\right]\times mode_{i}=\left(1-\frac{4}{7}\right)\times 6=2\frac{4}{7}$$

Using the new range for the remaining work, a new value of the estimated activity duration is generated. In this example, as 4 time units of work were already performed, the new minimum and maximum values are calculated as follows: $min_{2}=4+\frac{3}{7}=4\frac{3}{7}$, and $max_{2}=4+3\frac{3}{7}=7\frac{3}{7}$. In the case of the uniform distribution, the new duration value for Activity 2 will be randomized within the range $a_{2}\sim [U\frac{3}{7},\;7\frac{3}{7}$]. In the case of the beta distribution, in addition to the minimum and maximum values, the most likely value will be calculated as follows:$\;mode_{2}=4+2\frac{4}{7}=6\frac{4}{7}$. Using the new beta parameters, the new estimated duration generated from the updated distribution is $a_{2}\sim Beta\left(1\frac{3}{7},7\frac{3}{7},6\frac{4}{7}\right)$.

The above procedure is repeated for each realization and each potential inspection point, resulting in a list of possible project durations. Similar to how ${\tilde{H}}_{0}$ was evaluated, ${\tilde{H}}_{t}$ is calculated by using Equation (7), i.e., estimating all possible project durations at time t for each realization and calculating the empirical probability, $\tilde{p_{t}}$, of each range ∆t. The information gain per potential monitoring point can now be obtained, and in this specific example, the result is ${\tilde{I}}_{t}={\tilde{H}}_{0}-{\tilde{H}}_{t}=0.518-{\tilde{H}}_{t}$. Table 4 shows the output of a single simulation run and gives the information gain for each of the possible monitoring points. Note that the maximal information gain ${\tilde{I}}_{t}$ is obtained at monitoring point t=5 (bolded) in this simulation scenario; therefore, the recommended monitoring point for this run is selected as 5. A zero information value for t>6 implies that the realization does not change the estimation of the prior distribution of the project duration; thus, no new information is obtained with respect to the initial entropy ${\tilde{H}}_{0}$.

As the entropy at time t is calculated based on the specific realizations, a possible outcome can be ${\tilde{H}}_{t}\leq {\tilde{H}}_{0}$, resulting in ${\tilde{I}}_{t}\leq 0$ for a specific simulation (as in the case of t=2 in Table 4). This may occur in a single simulation wherein the common critical path has drastically deviated from the realization’s initial estimated value. From a practical perspective, a negative information value can result from how $p_{t}$ is estimated, i.e., by counting the number of occurrences in a specific range. Thus, it is possible that due to randomization effects, there might be cases where ${\tilde{p}}_{t}$ is skewed and leads to a value of ${\tilde{H}}_{t}$ that is lower than ${\tilde{H}}_{0}$. Such a situation represents an increased level of uncertainty at time t leading to a “loss” of information in comparison to t=0. Having many such cases reflects a situation wherein the initial distributions do not represent well the expected scenarios. Thus, a refinement of these initial distributions is needed. Note, however, that if the initial distributions are realistic and the simulations are executed multiple times, scenarios with negative information will hardly occur and thus will barely affect the optimal point selection, as seen below.

**Figure 3:**
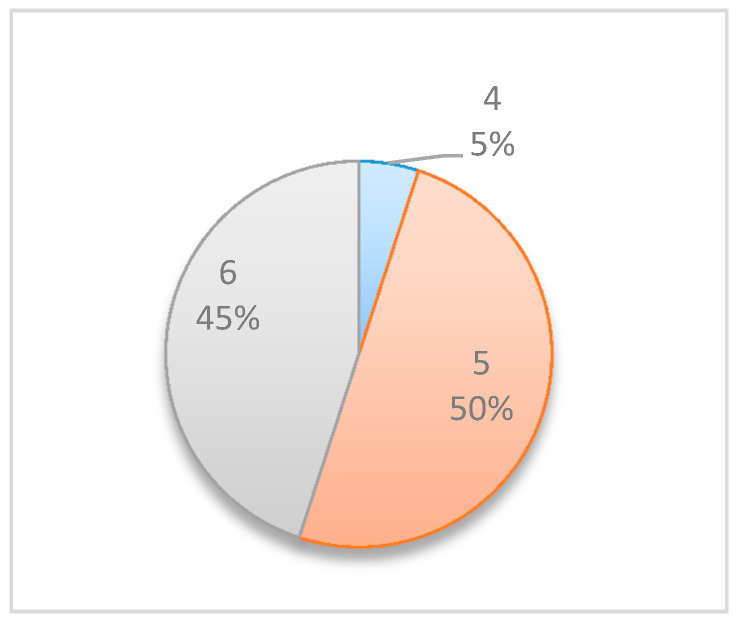
The illustrative running example: simulation results.

The above simulations are executed N  times. The output of the algorithm is, again, a table listing the selected optimal inspection points and the number of simulations in which each point was selected. For example, Figure 3 show the results of 10 simulations of the illustrative running example, in which monitoring points t=5 and t=6  were selected in 9 out of the 10 simulations as the optimum observation points for obtaining the maximal expected information gain. Further simulations can reveal that monitoring point  t=5 has a higher probability of resulting in the maximum information gain.

## 5. Numerical Experiments and Results

This section details the results of numerical experimentation using the above model. The experiments compare the proposed algorithm to other commonly used heuristics, using a variety of projects having different distributions and parameters. Various projects ranging from short projects with few activities to long projects with dozens of activities were examined. The experiments represent actual projects from various industries, such as IT and construction projects. Each experiment contained N=10/20 simulations per project with R=200 realizations per simulation. Both the uniform and beta distributions were used to examine the effects of the activity durations’ underlying distributions on the selected inspection points. The simulation model was implemented using VB.net, and the simulation runs were executed on a dual-core 8 GB PC.

### 5.1. Single Monitoring Point

#### 5.1.1. Example Project No. 1: Simple Project

**Figure 4:**
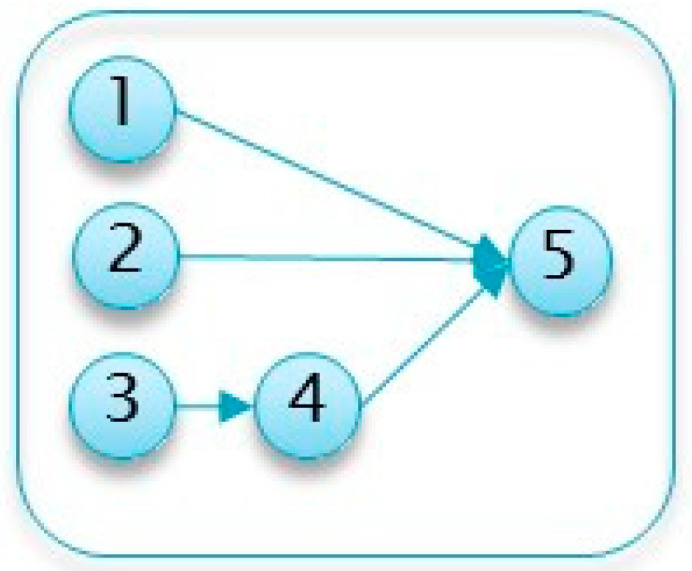
Example Project No. 1: Network Diagram.

The following project, detailed in Table 5 and Figure 4 follows the running example listed above and shows that even for relatively small-scale projects, it is not always trivial to identify the inspection point(s). Moreover, this project demonstrates that common heuristics based on the number of parallel activities or their variance can be misleading.

**Figure 5:**
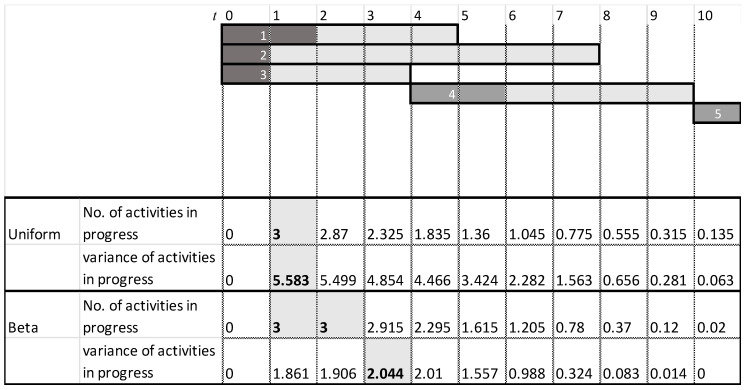
Example Project No. 1: heuristics.

**Figure 6:**
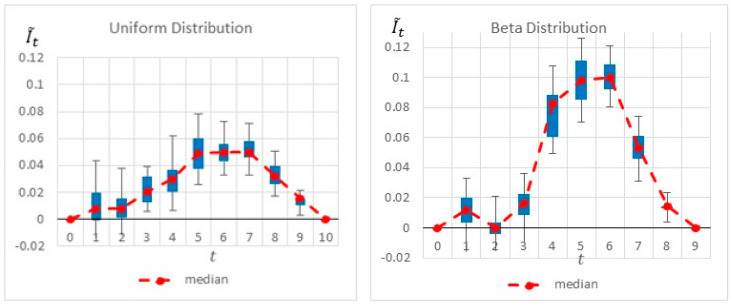
Example Project No. 1—Information Graph for a Single Monitoring point, (**left**) uniform vs. (**right**) beta.

Figure 5 shows the results of the heuristics as well as the project’s activities. The dark gray area of an activity represents the minimal activity duration, whereas the light gray area represents the elapsed time until reaching the maximal activity duration. The figure also shows the ‘worst-case scenario’ for which the critical path is maximal. The heuristics are calculated at the start of each time interval, and each heuristic’s maximal value is highlighted. As seen, common-practice heuristics locate the monitoring point at the beginning of the project, at t=1, where both the number of activities (3 activities) and the sum of their variances are the maximum. Intuitively, this appears to be a good inspection point, as it reveals information on 3 of the 5 activities. Thus, potentially more information can be gathered at this point. Note, however, that this is a relatively early stage of the project, for which most of the uncertainty related to the project duration remains the same based on the inspected activities. Moreover, as any of these three activities can impact the critical path, knowing that one or even two of them are progressing well does not necessarily affect the critical path’s duration, which is based on the maximal path. This intuition and logic are well reflected in the proposed information gain method: Although new information can be obtained at t=1, more information is gained by locating the monitoring point at a later time point, as indicated in Figure 6. The graph shows a slightly higher information gain at t=1, with a large variance due to the ongoing variances of the ongoing activities. Note, however, that a much higher information gain is obtained at t=5/6/7. This increase is mainly due to the fact that by t=5, Activities 1 and 3 are already completed, and the project manager is likely to have a better idea of the duration of Activity 2 and thus can better evaluate the project’s expected duration. Similarly, at *t* =6, in addition to the information gained on Activities 1, 2, and 3, the inspector obtains a better estimation of the duration of Activity 4, in turn making it easier to predict the critical path and the project duration.

**Figure 7:**
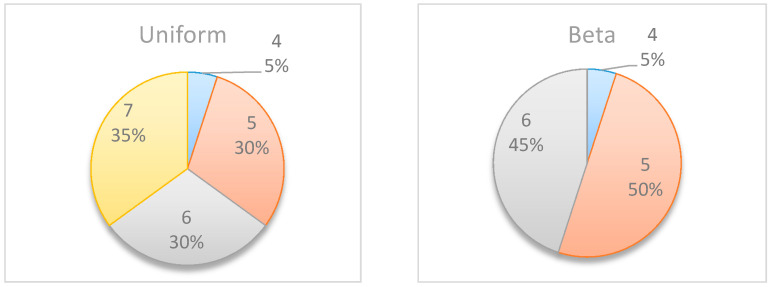
Example Project No. 1—Selected Single Monitoring point, (**left**) Uniform vs. (**right**) beta.

Figure 6 summarizes by box plot graphs the results of all simulations for both the uniform and beta distributions. The graphs show the amount of information gained, ${\tilde{I}}_{t}$, at each potential inspection point t. Figure 7 shows the distribution of the selected monitoring points over 20 simulations. Both figures demonstrate the importance of the “most likely” parameter, provided by the beta distribution, for the expected information gain and the selection of the monitoring points. As shown in the figures, the beta distribution results are more coherent in recommending t=5 or 6, whereas the uniform distribution results show an equal preference for inspection points at t=5, 6, or 7. This is an important outcome that justifies the additional overhead required by both the project manager and the team, enabling defining and maintaining additional information on the “most likely” value instead of merely providing the minimum and maximum values.

#### 5.1.2. The Effect of the “Most Likely” Parameter

In an attempt to further explore and understand the sensitivity of the model to the “most likely” value, Project No. 1 was slightly altered, and Activity 2′s “most likely” value was updated from 6 to 2. This new project is henceforth referred to as the ‘Beta B’ example.

**Figure 8:**
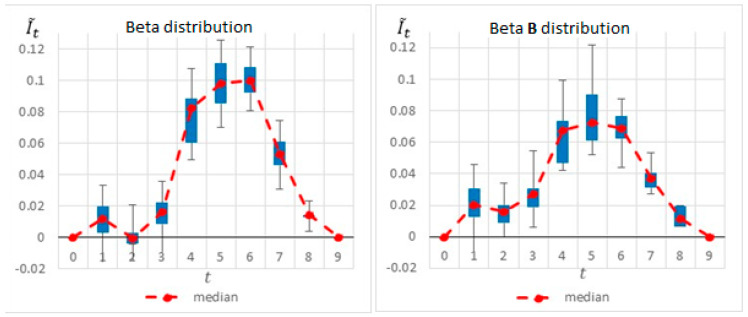
Simple Project No. 2: box plot graph for a single monitoring point, (**left**) Beta A vs. (**right**) Beta B distributions.

**Figure 9:**
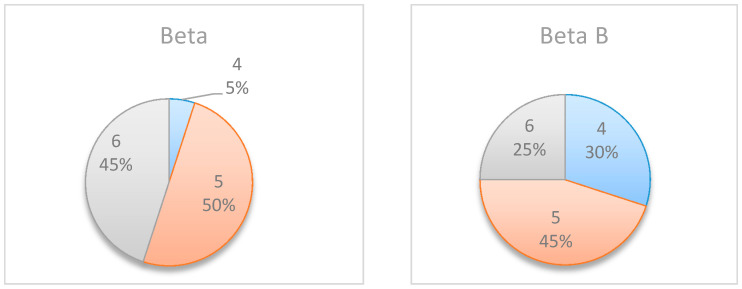
Simple Project No. 2: selected single monitoring point, (**left**) beta vs. (**right**) beta B.

The box plot graphs in Figure 8 and the pie charts in Figure 9 compare the results before and after the change, further demonstrating the importance of the “most likely” value and its effect on the selected inspection point. As shown in these figures, when shortening the duration of Activity 2, the potential information obtained at t=5\6\7 decreases, whereas the potential information obtained at t=2\3 increases. This is because Activity 2 is shorter, and therefore the project manager can learn more about the expected project duration earlier in the project.

#### 5.1.3. Underestimating the Project Duration

In this subsection, the example of Project No. 1 is further expanded to evaluate the proposed method in cases wherein the activities’ durations are underestimated. In this example, the realization times are deliberately modified such that they are longer vis-à-vis the planned times. Table 6 shows the differences between the original assumed minimal and maximal durations, which are used by the proposed method when re-evaluating the activities’ durations, versus the values actually used to generate the simulated realizations.

**Figure 10:**
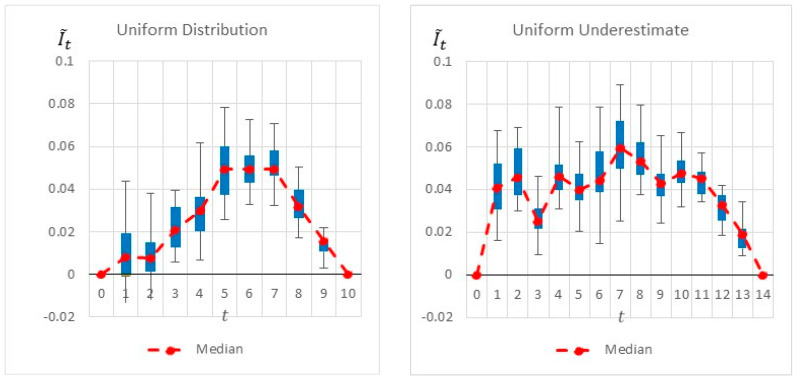
Simple Project No. 2: a box plot graph for a single monitoring point, (**left**) uniform vs. (**right**) an underestimated uniform distribution.

**Figure 11:**
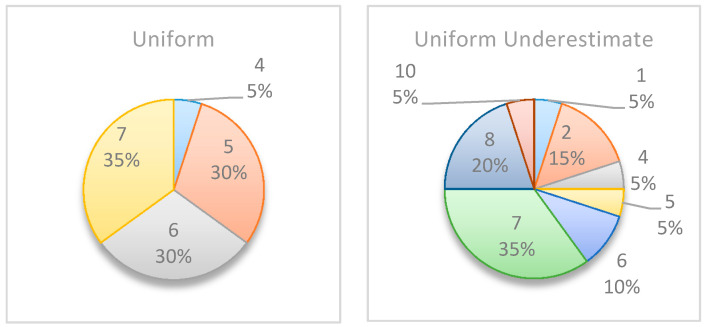
Simple Project No. 2: selected single monitoring point, (**left**) uniform vs. (**right**) underestimated uniform.

Figure 10 and Figure 11 compare the results of the original project to those of the underestimated project. The two figures clearly show that the model has readjusted the selected monitoring points and the potential information gain as a result of the realizations, which show that the project manager initially underestimated the activities’ durations. The box plot graphs and the pie charts are drastically changed from those of the original project. Based on the original project estimations, the monitoring points should have been located at t=5, 6, or 7. However, when using the underestimated project times, the selected points change, and while 5, 6, and 7 are still found to be relevant in some of the scenarios, new time points such as t=1, 2, 8, and 10 are now added as potential inspection points.

#### 5.1.4. Sensitivity Analysis: Number of Realizations

To better estimate the effect of the number of realizations on the accuracy of the selected inspection point, Example Project No. 1 is used with various numbers of realizations. In this experiment, R, the number of realizations, is increased from 200 to 300.

**Figure 12:**
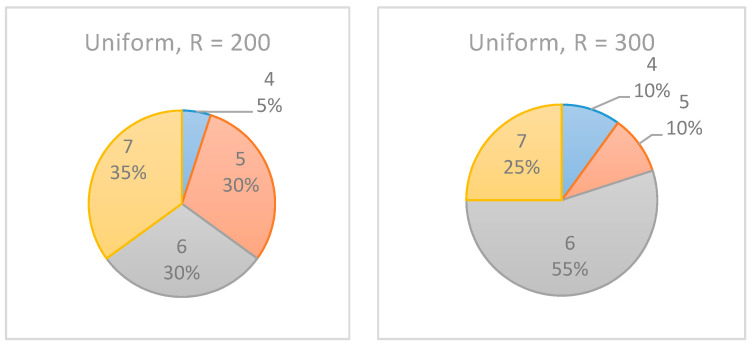
Example Project No. 1: selected single monitoring point, uniform dist. with (**left**) R = 200, (**right**) R = 300.

**Figure 13:**
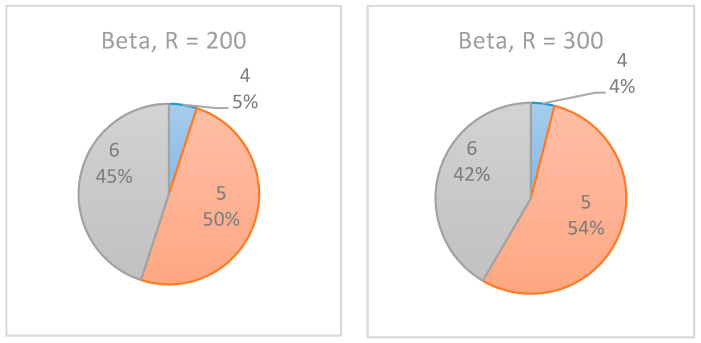
Example Project No. 1—selected single monitoring point, beta dist. with (**left**) R = 200, (**right**) R = 300.

As shown in Figure 12, the increase in the number of realizations affected the results only slightly, such that the confidence level of the recommended monitoring point increased. In the experiments with R = 200, the algorithm equally indicated 5/6/7 as the selected inspection point. However, when R was increased to 300 realizations, in 55% of the cases, t=6 was indicated as the preferred inspection point. Note, however, that for the beta distribution (see Figure 13), the same increase in the number of realizations had almost no effect on the results.

Accordingly, when using the proposed algorithm, if the results are not decisive, the project manager may want to increase the number of realizations to potentially obtain more consistent results. It may very well be that an increase will not affect the results, and in such cases, using the lowest value for R is recommended due to its effect on the algorithm’s run time.

#### 5.1.5. Example Project No. 2: Simultaneous Activities

The next medium-size project, given in Table 7, has more activities than the previous projects, and its network diagram is more complex.

**Figure 14:**
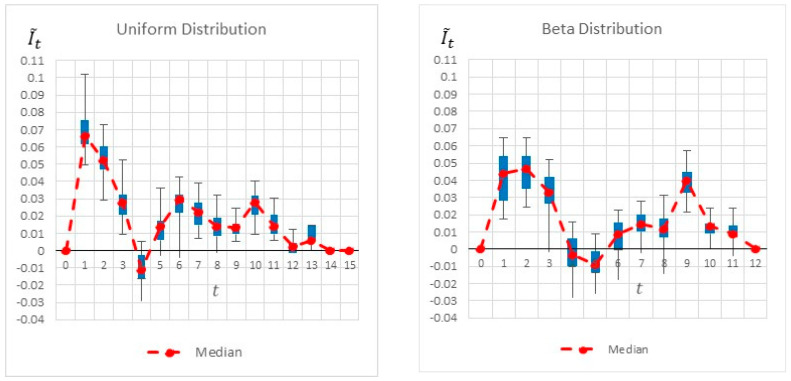
Example Project No. 2: box plot graph for a single monitoring point, (**left**) uniform vs. (**right**) beta distributions.

**Figure 15:**
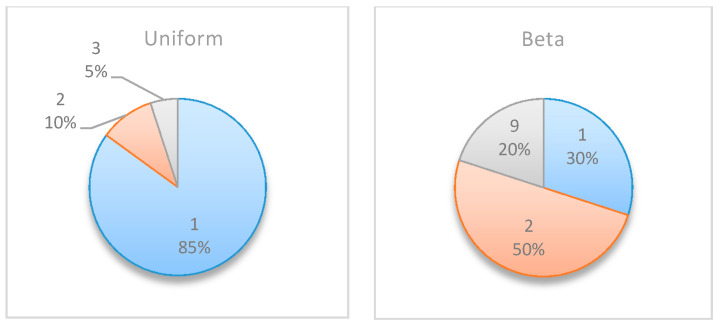
Example Project No. 2: selected single monitoring point, (**left**) uniform vs. (**right**) beta distributions.

The information gain graphs presented in Figure 14 are quite interesting and present a non-intuitive result. The information gain is not unimodal, i.e., it has some ups and downs, with potential information loss as time goes by. This observation contradicts the common notion that information gain should increase as a project progresses until the end of the project is near, when little uncertainty exists. As shown in Figure 14, there are time points that lead to a peak in the expected information gain, with a drop between these areas. In both information gain graphs, as well as in the pie charts presented in Figure 15, the proposed method shows a clear indication for locating the monitoring point at the beginning of the project. This result is due to the major effect of Activity 1 on the critical path, and thus on the overall project duration, as completing this activity is a prerequisite for moving on to the remaining activities. However, in later stages of the project, there are many more activities that are ongoing simultaneously, and their immediate and individual effects on the total project duration are not as high as that of the first activity. That is, if many activities are happening at the same time, there is a high enough probability that one or more of them will be delayed, causing the overall project duration to increase anyway. Another observable peak in the potential information gain can be obtained at t=9 (in the beta distribution), as by that time, Activities 1 to 7 are already completed and the remaining activities (8 to 13) are either ongoing or possibly completed as well, thus providing the project manager more clarity regarding the expected project duration.

#### 5.1.6. Example Project No. 3: Long, Complex IT Project

To evaluate the applicability of the proposed model in a real-life scenario, we used data collected based on a real IT deployment project that consisted of 112 stochastic activities with an estimated duration of approximately one year. The project network is relatively complex, and the critical path is frequently changing as time passes and the activities’ durations are updated. There is no doubt that a project manager would find it extremely difficult to identify the optimal monitoring points and is likely to use one of the common heuristics presented above (i.e., the maximum number of simultaneous critical activities).

**Figure 16:**
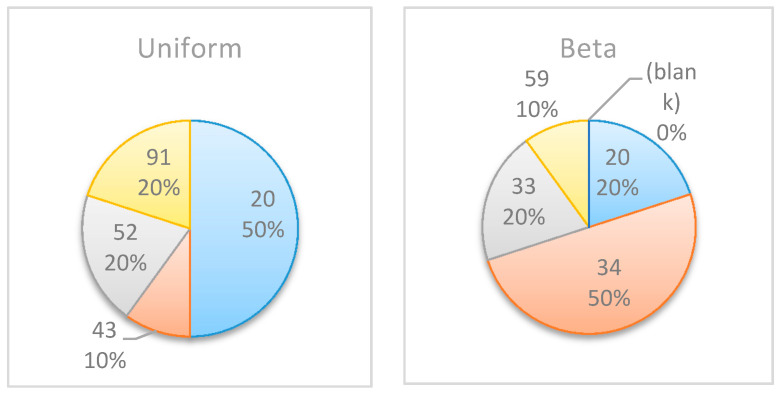
Example Project No. 3: Selected monitoring point: (**left**) uniform vs. (**right**) beta distribution.

The proposed algorithm was applied to the data, and despite the relative complexity, it identified the optimal monitoring points from 240 potential points. Figure 16 shows the selected monitoring points for both the beta and uniform distributions. As shown in this figure, the distribution type affects the outcome as well as the selected points, which are not necessarily similar, with the exception of a monitoring point at t=20, which is recommended by both cases and obtained the highest information gain in 50% of the simulations based on the uniform distribution and 20% of the simulations based on the beta distribution. This example shows again the complexity in evaluating the monitoring points in real-life projects a priori.

### 5.2. Multiple Monitoring Points

In many scenarios, there is a need and possibility of monitoring the project along its progression at several monitoring points. Note, however, that when looking for multiple inspection points, the brute force search problem with *K* monitoring points requires analyzing all possible combinations and can become too complex for a straightforward optimal policy. The experiments below show that often, when searching for multiple monitoring points, the previous monitoring points are found to be a subset of the set of final recommended points. For example, when searching for two points, one of the two recommended monitoring points is one of the selected monitoring points when searching for a single point. This observation can be used to propose a simple heuristic that reduces the computational complexity of the algorithm for multiple monitoring points and to narrow the search domain such that when searching for K points, the K−1 previous search points are used, and only the Kth point has to be located. Such a heuristic is described in Figure 1.

In this numerical study, further experiments were conducted to allocate multiple monitoring points for the project management examples above (mainly, Example Project No. 1 shown in Table 5 and Example Project No. 2 shown in Table 7). Table 8 compares the obtained results when searching for two monitoring points vs. those obtained when searching for a single monitoring point. For each project, the table shows the selected points and the corresponding percentages of the simulations in which those points were selected as those expected to generate the highest information gains. For example, in Simple Project No. 1 with a beta distribution, when searching for a single monitoring point, the results were {5 [50%], 6 [45%], and 4 [5%]}, implying that t=5 was selected as the optimal monitoring point in 50% of the simulations, t=6 was selected in 45% of the simulations, and t=4 was selected in 5% of the simulations.

In all of the experiments, when searching for two monitoring points, one of the two selected points was identified in the search for a single monitoring point as an optimal point (these points are bolded in the last column of the table). For example, in Experiment No. 4, when searching for two monitoring points in Example Project No. 2 with a beta distribution, in all of the results, one of the two recommended monitoring points is located at t=1, 2 or 9, which is one of the selected points when searching for a single monitoring point. However, note that the point that was most frequently selected when searching for a single monitoring point may not be as frequently selected when searching for two monitoring points. For example, in the same experiment (No. 4), while t=2 was the most frequently selected point when searching for a single point, it only appeared in 35% of the cases when searching for two monitoring points, while t=1, which had a lower frequency when searching for a single monitoring point, was recommended in 70% of the cases when searching for two monitoring points. Nonetheless, in cases where the search for a single monitoring point resulted in a very high preference for a specific point, a similar preference was observed in the search for two monitoring points. For example, in Experiment No. 3, t=1 was selected in 85% of the cases when searching for a single monitoring point, and t=1 was also one of the selected points in 95% of the results when searching for two monitoring points.

This observation leads to a simple heuristic that can narrow the search domain when looking for multiple monitoring points, so that one of the two monitoring points will be taken from the set of points that were already selected when searching for a single monitoring point. To further test the above outcomes, another experiment, shown in Table 9, was conducted to search for three monitoring points for Example Project No. 1. As shown in Table 9, when searching for three monitoring points, one of the points in this example is always one of the recommended points obtained by the search for two monitoring points. Note that while two of the points are recommended when searching for two monitoring points, the combination of the two may not be optimal.

For example, in Experiment No. 2, the combination (6,7,10) was recommended by 10% of the simulations when searching for three monitoring points under a beta distribution. Note that none of the subset combinations, i.e., (6,7), (7,10), or (6,10), was recommended by the simulations when searching for two points. However, monitoring points t=6 and t=7 were selected in combination with other points, e.g., (5,6) and (5,7). This observation again implies that the heuristic suggested above may be used to narrow the search domain, yet without guaranteeing a global optimization solution.

## 6. Conclusions

In this paper, an information-theoretic approach is proposed for identifying potentially good project inspection points. The objective of the inspection is to better estimate a project’s overall duration by a given number of inspection points. Accordingly, the proposed approach measures the expected information gain about the estimated project duration at each potential inspection point and selects the most informative inspection points. Previous studies that also aimed to obtain informative monitoring points considered, for example, some simplifying assumptions regarding project activities [5], partially related heuristics taken from electromagnetism theory [11], and an intensity measure of the activities as an indirect indication of the anticipated information gain [5]. Other practical approaches did not use a clear mathematical formulation to address the monitoring optimization problem and instead proposed locating the inspection points by practical rule-of-thumb measures, such as the ‘number of activities in progress’ or ‘total variance of ongoing activities’. These methods were shown to result in non-optimized inspection points in terms of their expected information gain. Moreover, we show by just a few examples that popular rule-of-thumb practices can result in non-informative outcomes, such as locating the monitoring points at times with many activities running in parallel, where the information gathered provides (at that point) a poor estimation of the overall project’s duration, e.g., there is a high probability that, even if almost all activities are progressing as expected at that time, one or more activities will be delayed later, causing the overall project duration to be extended. In any case, none of the above methods applied a straightforward, systematic, information-theoretic approach to the project plan as proposed herein.

Several experiments were conducted on multiple projects varying in length, size, and complexity to analyze the model’s output. Each project was studied by using both the uniform distribution, which is the maximal entropy distribution when only the minimal and maximal activity durations are known, and the beta distribution, which is commonly used to represent projects’ activity durations. The effect of the beta distribution’s ‘most likely’ value on the information gain suggests that the overhead required to obtain and maintain information on such a value is justified, as it often leads to finding optimal inspection points. This phenomenon was further demonstrated by comparing two identical projects both following a beta distribution in terms of their activity duration, yet with differing ‘most likely’ values.

The proposed algorithm can be applied to find a single monitoring point as well as a combination of monitoring points. In terms of complexity, it is mainly sensitive to the maximal length of the project and the required number of monitoring points and not necessarily to the number of activities. Based on the experimental results, a heuristic was proposed to reduce the algorithm’s complexity when searching for K monitoring points by using the results of the K−1 previous points to narrow the search domain significantly. In addition, one can reduce the complexity by changing the granularity of time units, for example, by searching for the optimal control weeks instead of the optimal control days. This allows for solving large problem instances and finding an optimal inspection protocol for long and complex projects in a shorter run time.

Future research may include investigating the effectiveness of the suggested multiple control point heuristics vs. the greedy approach, as the suggested method is still computationally challenging for guaranteeing multiple optimal control points. The study of the effects of various time scales and discretization methods on the obtained information can shed more light on the robustness of the method. Another direction is to expand the proposed model to address not only the information gain for the overall project duration but also the information’s applicability and ability to be used to overcome potential risks and delays. This last point is also related to our activity independence assumption, which implies that, at inspection point *t*, any information about the ongoing or completed activities is not considered relevant for estimating the duration of future activities at that time. This is a limiting assumption since there are cases where the information gathered on the ongoing or completed activities at an inspection point can be used to re-estimate the duration of related future activities. For example, if future activities involve similar resources, tasks, or teams, then an earlier/later completion of related ongoing or completed activities could provide valuable insights into their duration.

## Figures and Tables

**Table 1:** The illustrative running example: a project with beta-distributed activities.

Activity (i)	Predecessors	Min Duration (*min_i_*)	Max Duration (*max_i_*)	Likely Duration (*mode_i_*)
1		2	5	4
2		1	8	6
3		1	4	3
4	3	2	7	3
5	1,2,4	1	1	1

**Table 2:** The illustrative running example: realization values based on the underlying beta distributions.

Realization (r)	$a_{1}$	$a_{2}$	$a_{3}$	$a_{4}$	$a_{5}$
1	4.68	5.85	3	3.83	1
2	3.77	7	2.83	4.11	1
3	4.18	4.87	2.36	2.82	1
4	3.41	4.55	3.41	5.95	1
5	4.3	7.23	3.24	2.52	1
6	3.5	7.24	2.94	5.04	1
7	4.27	4.55	3.18	3.33	1
8	3.92	4.34	3.14	4.77	1
9	4.33	5.07	2.78	3.95	1
10	2.89	5.55	3.33	2.84	1

**Table 3:** The illustrative running example: possible project duration realizations.

Realization (r)	$z_{1}$	$z_{2}$	$z_{3}$	Critical Path	$x_{r}$
1	5.68	6.85	7.83	$Z_{3}$	7.83
2	5.18	8	7.94	$Z_{2}$	8
3	4.77	5.87	6.18	$Z_{3}$	6.18
4	4.41	5.55	10.36	$Z_{2}$	10.36
5	5.3	8.23	6.76	$Z_{2}$	8.23
6	4.5	8.24	8.98	$Z_{3}$	8.98
7	5.27	5.55	7.51	$Z_{3}$	7.51
8	4.92	5.34	8.91	$Z_{3}$	8.91
9	5.33	6.07	7.73	$Z_{3}$	7.73
10	3.89	6.55	7.17	$Z_{3}$	7.17

**Table 4:** The illustrative running example: entropy and information gain per monitoring point.

t	${\tilde{H}}_{t}$	${\tilde{I}}_{t}$
0	0.518	0
1	0.473	0.045
2	0.639	−0.120
3	0.533	−0.015
4	0.447	0.071
5	0.348	**0.170**
6	0.408	0.110
7	0.518	0
8	0.518	0
9	0.518	0
10	0.518	0
11	0.518	0
12	0.518	0

**Table 5:** Example Project No. 1: Activity details.

Activity (*i*)	Predecessors	Min Duration (*min_i_*)	Max Duration (*max_i_*)	Most Likely (*mode_i_*)
1		2	5	4
2		1	8	6
3		1	4	3
4	3	2	7	3
5	1,2,4	1	1	1

**Table 6:** Project No. 2 Activities: project underestimation, new range.

Activity (i)	Predecessors	Assumed Min Duration	Assumed Max Duration	Actual Min Duration	Actual Max Duration
1		2	5	3	7
2		1	8	4	10
3		1	4	3	6
4	3	2	7	5	9
5	1,2,4	1	1	1	1

**Table 7:** Example Project No. 2: activity details.

Activity (*i*)	Predecessors	Min Duration (*min_i_*)	Max Duration (*max_i_*)	Most Likely (*mode_i_*)
1		2	5	3
2	1	1	5	3
3	1	1	3	1.5
4	1	2	4	3.5
5	1	1	3	2
6	1	1	3	1.5
7	1	2	5	4
8	2,5	1	3	2
9	2,3	1	3	2.5
10	3,4	1	4	2
11	3,6	1	3	2
12	2,7	1	3	1.5
13	2,4	1	3	2.2
14	8,9,10,11,12,13	1	2	1.7

**Table 8:** Single vs. two monitoring points: summary of results.

#	Project	Distribution	Single Monitoring Point	Two Monitoring Points
1	Example Project No. 1 (Table 5)	Uniform	{7 [35%], 6 [30%], 5 [30%], 4 [5%]}	{(**5**,**6**) [20%], (**5**,**7**) [10%], (**5**,8) [10%], (**6**,7) [10%], (**6**,12) [10%], (**5**,10) [5%], (**6**,8) [5%], (**6**,9) [5%], (**7**,8) [5%], (**7**,9) [5%], (2,**5**) [5%], (2,**7**) [5%], (4,**7**) [5%]
2		Beta	{5 [50%], 6 [45%], 4 [5%]}	{(**5**,**6**) [45%], (**5**,7) [20%], (**5**,8) [10%], (**5**,9) [5%], (**5**,11) [5%], (**4**,9) [5%], (**4**,11) [5%], (**6**,11) [5%]}
3	Example Project No. 2 (Table 7)	Uniform	{1 [85%], 2 [10%], 3 [5%]}	{(**1**,**3**) [25%], (**1**,6) [15%], (**1**,**2**) [10%], (**1**,15) [10%], (**1**,9) [10%], (**1**,5) [5%], (**1**,7) [5%] (**1**,10) [5%], (**1**,11) [5%], (**1**,12) [5%], (**2**,7) [5%] }
4		Beta	{2 [50%], 1 [30%], 9 [20%]}	{(**1**,9) [20%], (**2**,9) [15%], (**1**,14) [15%], (**1**,12) [10%], (**2**,8) [10%], (**1**,**2**) [5%], (**1**,3) [5%], (**1**,5) [5%], (**1**,7) [5%], (**1**,11) [5%], (**2**,7) [5%]}

**Table 9:** Three Monitoring Points Results: Example Project No. 1.

#	Distribution	Single Monitoring Point	Two Monitoring Points	Three Monitoring Points
1	Example Project No. 1 with a normal distribution (Table 5)	{7 [35%], 6 [30%], 5 [30%], 4 [5%]}	{(5,6) [20%], (5,7) [10%], (5,8) [10%], (6,7) [10%], (6,12) [10%], (5,10) [5%], (6,8) [5%], (6,9) [5%], (7,8) [5%], (7,9) [5%], (2,5) [5%], (2,7) [5%], (4,7) [5%]}	{(6,7,8) [30%], (6,7,11) [15%], (6,7,9) [10%], (6,7,10) [10%], (6,7,12) [10%], (3,5,7) [5%]), (5,9,12) [5%]), (6,10,11) [5%]), (7,8,12) [5%]), (7,8,9) [5%])}
2	Example Project No. 1 with a beta distribution (Table 5)	{5 [50%], 6 [45%], 4 [5%]}	{(5,6) [45%], (5,7) [20%], (5,8) [10%], (5,9) [5%], (5,11) [5%], (4,9) [5%], (4,11) [5%], (6,11) [5%]}	{(6,7,10) [15%], (5,8,9) [10%], (5,8,10) [10%], (5,8,11) [10%], (6,7,8) [10%], (6,7,9) [10%], (5,10,11) [5%], (5,10,12) [5%], (5,6,12) [5%], (5,8,12) [5%], (6,8,9) [5%], (6,9,12) [5%], (6,11,12) [5%]}
